# Epidemiology, diagnosis and emerging therapies for Lyme disease of the Northern Hemisphere

**DOI:** 10.1186/s12245-026-01182-5

**Published:** 2026-04-11

**Authors:** Shairy Priya, Sathvik Belagodu Sridhar, Javedh Shareef, Tarun Wadhwa, Balamurugan Balusamy, Dhanalekshmi Unnikrishnan Meenakshi, Sonali Sundram, Rishabha Malviya

**Affiliations:** 1 Department of Pharmacy, School of Medical and Allied Sciences, Galgotias University, Greater Noida, U.P 201308 India; 2 RAK College of Pharmacy, RAK Medical & Health Sciences University, Ras Al Khaimah, United Arab Emirates; 3 Department of Pharmacy Practice, College of Pharmacy, Dubai Medical University, Dubai, UAE; 4 School of Engineering and IT, Manipal Academy of Higher Education, Dubai Campus, Dubai, UAE; 5 College of Pharmacy, National University of Science and Technology, Muscat, Oman

**Keywords:** Epidemiology, Pathogenesis, Biomarkers, Neuroinflammation, Prevention

## Abstract

**Background:**

Lyme disease is the most widespread tick-borne infection in the Northern Hemisphere and is challenging to diagnose and treat due to its changing clinical presentation, antigenic variation, tissue tropism, and the expanding distribution of vectors. This review includes ecology, pathogenesis, diagnostics, treatment, post-treatment, prevention, and novel translational approaches.

**Methods:**

A literature review was conducted to include literature published between January 2000 and March 2026 in PubMed/MEDLINE, Scopus, and Web of Science, with landmark studies used where applicable. Original research, clinical trials, systematic reviews, and major public health reports were prioritised.

**Results:**

Two-tier serology is the most common diagnostic technique, but it has limited sensitivity in early infection and does not distinguish between active and past infection. Culture and PCR are only useful in a few instances. The use of new technologies such as multiomics biomarkers, metagenomics, T-cell assays, and AI-enhanced diagnostics is promising but has not yet been tested in a prospective multicentre study. Most of the early and disseminated disease can be treated with standard antibiotics, whereas the long-term therapy of PTLD is not justified and can cause more adverse effects. These preventive and curative advancements involve VLA15 vaccination, anti-tick and reservoir-specific approaches, microbiome-engineered vectors, and anti-persister/ biofilm.

**Conclusion:**

Lyme disease requires combined prevention, improved diagnostics, enhanced biomarker research, and well-designed PTLD trials. The short-term benefits will be based on the optimisation of existing diagnostics and vector control, and the long-term benefits will be based on rigorous validation of vaccines, biomarkers, and specific therapies.

## Abstract (fr)

**Clinical trial number:**

Not applicable.

**Graphical Abstract:**

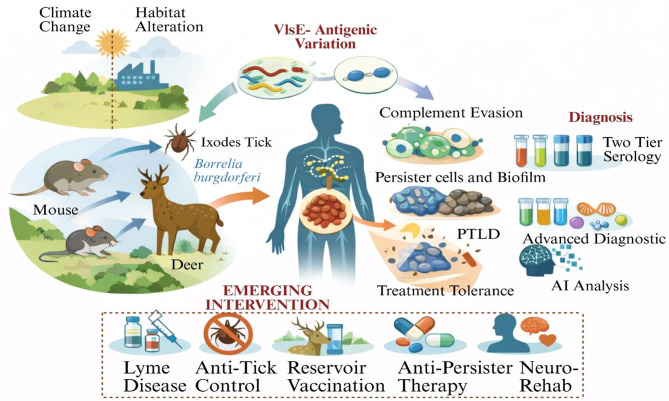

## Introduction

*Borrelia burgdorferi* sensu lato, a spirochete, causes Lyme disease, the most prevalent vector-borne disease in the Northern Hemisphere, which is temperate. It is transmitted by *Ixodes spp*. ticks [1]. The surveillance of the United States alone documented a bit more than 89,000 reported cases in 2023; by comparison, estimated incidence through models of about 476,000 treated cases annually incorporates under-ascertainment and relies on alternative data and approaches (e.g. analysis of insurance claims and electronic health records, capture-recapture or multiplier techniques and sensitivity to diagnostic underestimation and latent cases) [2]. Since passive surveillance records only cases that have been confirmed in labs or formally reported, whereas modelled estimates include clinically diagnosed and treated cases, along with statistical adjustments for underreporting, the latter is likely to be higher than routine surveillance numbers and more reflective of the actual clinical burden [1].

Clinically, it ranges from variably localised erythema migrans rash and flu-like disease to disseminated infection involving joints, heart, and central nervous system; heterogeneity of symptoms and overlap with other multisystem syndromes pose a barrier to early or accurate diagnosis, and heterogeneity of patient outcomes [3].

Despite decades of research, existing reviews tend to address ecological, molecular, diagnostic, and clinical factors separately. This siloing constrains the ability to produce impact across the disciplines, since the complex of Borrelia transmission (ecology), pathogenic bio-phenotype (antigenic variation and tolerance), host responses (immune and neuroinflammatory), and diagnostic biomarkers (serology, nucleic acid tests, and novel omics signatures) are closely linked. As an illustration, climate-promoted growth of Ixodes areas alters exposure risk and reservoir-community make-up, which subsequently modulate pathogen genetic variation and human illness phenotypes, providing a holistic understanding that is scarcely analogically amalgamated in the extant literature [1, 4].

In this review, an ecology-omics-clinical perspective is adopted, extending previous multi-disciplinary studies but focusing more specifically on translational aspects. Previous integrative reviews have focused on a single component of tick ecology (e.g., microbiomes or omics biomarkers) or on a pair of them (e.g., with clinical management). They explicitly synthesise these domains, identifying or exploring translational entry points in the interaction between tick ecology and their microbes through tick and reservoir interventions and molecular discovery through multi-omics and host-response methods toward clinical implementation, including trial design, diagnostic pipelines, and regulatory considerations. First, the tick microbiome is highlighted as a potential intervention target; recent metagenomic and microbiome research reveals complex, non-core microbiomes in tick vectors and suggests opportunities to reduce tick vector competence through microbiome surveillance or engineering [5, 6]. Second, the combination of multi-omics approaches (transcriptomic, proteomic, metabolomic, lipidomic) with AI-integrated diagnostic pipelines emphasises not only knowledge demonstration but also focuses on validation, standardisation, and regulatory actions to bridge the gap in two-tier serology and differentiate active infection from post-infectious or immune-mediated diseases [7, 8].

Third, biofilm-like training is observed in mechanistic studies, indicating biofilm-like aggregates and persister cells in Borrelia, thereby demonstrating antibiotic tolerance. This provides a plausible explanation for recalcitrant courses, but direct causal links to PTLD in humans are still unproven. Therefore, a set of anti-persister strategies should be considered as preclinical or investigational, pending further evidence from translational research [9, 10]. Fourth, evidence supports the neuroinflammation in some of the patients having continuous symptoms of cognitive and fatigue following the treatment; candidate biomarkers such as molecular imaging (e.g., TSPO PET) and CSF cytokine profiles can be used to stratify the patients to receive specific non-antibiotic therapies [11, 12]. Finally, prevention strategies are gaining momentum with next-generation vaccine candidates. Multi-valent OspA-based vaccine candidate VLA15 is in a late-stage (Phase 3) clinical trial; its safety and efficacy are under review, and regulatory approval depends on the trial results. Such vaccines, once proven to be safe and effective, have the potential to add a scalable primary prevention focus back to the portfolio and complement more comprehensive One Health solutions, such as antitick and reservoir-specific interventions which combine human, veterinary and ecological solutions [3, 13].

The present manuscript was written as a narrative review. PubMed/MEDLINE, Scopus, and Web of Science databases were used to search for articles published between January 2000 and March 2026; landmark studies were included where applicable. The search terms included Lyme disease, *Borrelia burgdorferi, Ixodes,* post-treatment Lyme disease, diagnostics, multi-omics, artificial intelligence, persister cells, tick microbiome, vaccines, and One Health. Original research papers, clinical trials, systematic reviews, and large-scale public health reports were given priority when peer-reviewed. Case reports were not included, as well as non-peer-reviewed opinion articles, unless they were explicitly related to a growing or controversial issue. In case there was a lack of sufficient evidence or a heterogeneous set of findings was identified, they were interpreted with caution and areas of uncertainty were mentioned explicitly, which served to minimise bias and explain the evidentiary strength.

## Epidemiology and global distribution of *Borrelia burgdorferi sensu lato* infections

**Fig. 1:**
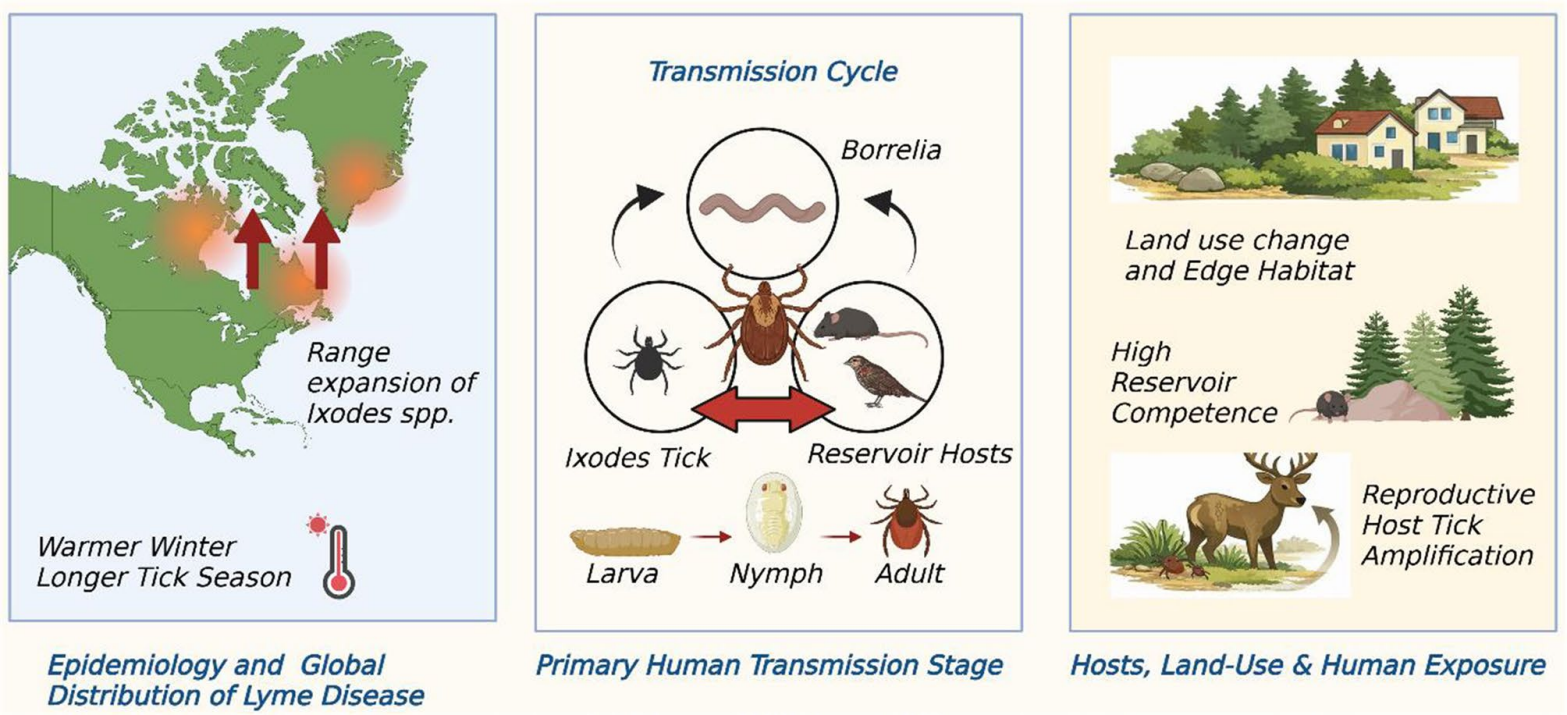
Land-use change, reservoir hosts, and tick amplification increase human risk for *Borrelia burgdorferi* sensu lato infections (Lyme borreliosis in regions where multiple *B. burgdorferi* genospecies circulate)

The ecological nature of the spatial distribution and the public-health impact of Lyme disease are as follows: the risk is defined by the triad of *Borrelia spp*., Ixodes tick vectors, and vertebrate reservoir hosts (all of which are dynamically restructured by human behaviour, climate, and land use). There is a consistent set of records over the past 20 years regarding the northward and altitudinal range shifts of various *Ixodes spp*. in temperate areas, attributed to surging temperatures and extended seasons that allow tick development and support their questing behaviour [14, 15]. It is an interaction between climate-induced changes, including warmer winters, earlier springs, and changed humidity regimes, and fragmentation of forest and peri-urban patches to enhance contact between humans and ticks in suburban and exurban environments, that causes locally high incidence of Lyme disease despite overall habitat loss to urban growth and development [16, 17]. Climate warming, land-use change, reservoir competence, and host amplification collectively shape the spatial and temporal dynamics of Lyme disease transmission, as shown in Fig. 1.

### Global incidence and prevalence

Epidemiologic burden estimates must be interpreted within the context of regional diagnostic capacity, surveillance design, and case-definition harmonisation, which vary substantially across continents. Entomological surveys and mechanistic modelling of alpha scales of blacklegged ticks (*Ixodes scapularis*) and related species have authors who now support suitability envelope surfaces for climate variables (temperature, accumulation of degree-days, relative humidity) and host availability [14, 15, 18]. Such studies demonstrate that not only does the possible area that competent vectors can occupy under recent warming conditions also increase, but in many locations, such as those, the seasonality of nymphal activity, the stage most closely linked with human transmission, is lengthened, providing humanity with increased exposure time [18]. The exposure is enhanced by land-use change and suburbanization, which create edge habitat where small-mammal reservoirs flourish, as well as by people’s recreation or even living in areas with tick habitat [16].

The increasing incidence, geographic expansion, and regional variability of Lyme disease are summarised in Table 1.

**Table 1:** Global Epidemiology of Lyme Disease/Lyme borreliosis and Key ecological drivers

Geographic region	Dominant Borrelia species	Primary Ixodes vector	Estimated disease burden	Major ecological drivers	References
North America (USA, Canada)	*B. burgdorferi* sensu stricto	*Ixodes scapularis*, *I. pacificus*	Approx. 476,000 treated cases/year in the USA	Climate warming, suburbanisation, forest fragmentation, high deer and rodent density	[1, 14, 16, 18]
Western and Central Europe	*B. afzelii*, *B. garinii*	*Ixodes ricinus*	Incidence varies by country; in endemic areas up to tens-hundreds per 100,000/year	Milder winters, extended tick activity seasons, peri-urban green spaces	[15, 17, 19]
Northern and Eastern Europe	*B. afzelii*, *B. garinii*	*Ixodes ricinus*	Expanding geographic range; variable national incidence	Temperature rise, land-use change, and wildlife host shifts	[15, 17]
Asia (China, Mongolia, Russia)	*B. garinii*, *B. afzelii*	*Ixodes persulcatus*	Confirmed vector presence and regional human cases; surveillance heterogeneity limits precise burden estimates	Climate suitability, limited surveillance, wildlife reservoirs	[17, 20]

In global epidemiologic interpretation, distinguish the ecological suitability, recorded presence of vectors, serologic human exposure approximations, and clinical prevalence of Lyme borreliosis (“Lyme borreliosis” is used for regional discussions in Europe and Asia where multiple *Borrelia burgdorferi sensu lato* genospecies (for example, *B. afzelii*, *B. garinii*) contribute to human illness. The term “Lyme disease” is retained for North American contexts where *B. burgdorferi* sensu stricto predominates. This distinction clarifies taxonomic and clinical differences relevant to diagnostics, ecology, and public-health practice. Normalised surveillance solutions and molecular confirmation have yielded fairly robust incidence estimates in North America and Europe, largely attributable to *B. burgdorferi* sensu stricto in North America and to *B. afzelii* and *B. garinii* in Europe. Conversely, in some regions of Asia and the Middle East, the data is heterogeneous and is usually based on entomological surveys, regional seroprevalence projects or case series in the hospitals instead of population-level surveillance. In China and Mongolia, e.g. Ixodes persulcatus is established and several *B. burgdorferi sensu lato genospecies have been identified, although nationally standardised* incidence reporting is not yet in place. Equally, serologic evidence and ecologic suitability are reported in India and parts of the Middle East, but are not harmonised by diagnostic criteria or based on longitudinal confirmation of cases. Consequently, reportage of “expansion/increasing burden in regions with a limited history of study should be viewed in light of better detection, new surveillance arrangements, and better ecological mapping as opposed to the presumed growth of incidence as a unit.

### Reservoir hosts and transmission network

Host community composition determines the dynamics of transmission in two ways: reservoir competence (the capacity to infect feeding ticks) and tick population amplification (the capacity to sustain or increase tick reproduction and abundance). In northeastern North America, the white-footed mouse, *Peromyscus leucopus*, is a very efficient reservoir of *Borrelia burgdorferi* sensu stricto, effectively transmitting the pathogen to larval ticks and thus maintaining the infection’s prevalence. Also, locally small mammals and some bird species may be involved in pathogen maintenance through infecting feeding ticks [19, 21]. Conversely, white-tailed deer are not considered good reservoirs of *B. burgdorferi* and do not significantly contribute to the pathogen’s transmission, whereas populous ticks rely on adult Ixodes ticks as their reproductive hosts, directly affecting population density. The empirical study reveals that the correlation between the number of deer and human Lyme risk depends on context and is driven by the relationship between tick abundance and the prevalence of infections in competent reservoir hosts [22, 23]. Pet-owners and synanthropic wildlife also play a further role in updating peri-domestic exposure risk and in demonstrating the significance of understanding host utilisation at various stages of the tick life cycle (larva, nymph, adult) to shape specific interventions [19].

### Rare/Novel integration: tick microbiome engineering

The tick microbiome is a novel and under-researched regulator of Lyme disease transmission. More recent high-resolution 16S rRNA and metagenomic surveys have shown complex microbial populations within the midgut and salivary glands that engage the host and have the ability to regulate tick vector competence [5, 24]. Specific microbial taxa and community configurations have also been found by experimental and correlational studies to either prevent or promote colonisation of the tick gut and the salivary glands by Borrelia to compose the conceptual framework of microbiome-based biocontrol strategies [5, 25]. Importantly, outside descriptive surveys and more-or-less speculation on microbiome engineering, it is currently experimentally demonstrable that host immunisation against targeted tick-associated microbiota can modify the midgut microbial community composition, shape tick immune responses, and dramatically decrease the colonisation of *Ixodes ricinus* by *Borrelia afzelii* on the basis of microbiota perturbation in a One Health system [25–28].

Nevertheless, it is unclear how microbiome manipulation, such as vaccination, paratransgenesis, and CRISPR-based symbiont editing, can ever be applied at the field scale, and it poses significant challenges in terms of feasibility and ecological cost. They are the realisation of stable, consistent microbial changes in natural tick populations, preventing unwanted off-target ecological shifts or horizontal transfers, and the limitations of regulation and its acceptance by society. Even though technical feasibility has been demonstrated in controlled models, regulatory, ecological, and social barriers remain significant impediments to deployment in the field [25, 28]. Based on this, the tick microbiome engineering approach must be discussed as a long-term, investigational method that requires gradual ecological hazard assessment and effective oversight, rather than an intervention that assumes deployment.

### Predictive modelling and GIS-based risk maps

Lyme risk mapping and forecasting are increasingly integrated using GIS, remote sensing, and machine learning to generate high-resolution risk surfaces that combine climate, land cover, and host-density proxies with entomologic surveillance. Its practical uses include locating hotspots for acaricide deployment, educating the public, and ranking locations for implementing reservoirs or anti-tick programs. However, most models lack prospective or geographically independent validation, limiting claims of operational forecasting capability.

These accuracy claims must be moderated: most studies evaluate model performance using internal cross-validation or retrospective out-of-sample tests rather than external validation or prospective application. Therefore, statements about operational or near-real-time predictive capabilities should be externally or prospectively validated; only models demonstrated to be predictive in independent settings or through prospective studies can be considered operationally predictive. Another avenue for expanding official surveillance beyond top-down modelling includes community-scale surveillance, such as citizen drag-sampling schemes, smartphone applications reporting tick bites, and public reporting of erythema migrans. Citizen science approaches that integrate these methods provide greater sensitivity but require bias correction, calibration, and independent validation before they can be used for public health decision-making [16, 29]. Combined, these geospatial and community-innovative tools facilitate precision public health: implementing interventions at locations where ecological risk is elevated, maintaining a perspective on the impacts of landscape-level alterations, and providing early alerts of the substantiation of new Lyme hotspots.

## Borrelia biology, persistence and pathogenesis

### Antigenic variation and immune evasion

One of the most essential ways of evading host adaptive immunity is antigenic variation of the outer proteins in *B. burgdorferi.* The variant primary protein-like sequence expressed (VlsE) system provides antigenic diversity by recombining the Vls locus, enabling dynamic changes in VlsE epitopes that overcome antibody recognition and clearance immunity during persistent infection [30, 31]. Recent structural and sequence studies point to the complexity of Vls antigenic variation systems in Borrelia genospecies and their evolutionary optimism to evade immune responses [32].

*B. burgdorferi* also has complement evasion mechanisms that overcome innate immunity in addition to antigenic variation. The binding proteins, such as OspE, CspA, and CspZ, bind host complement regulators on the bacterial surface and prevent the activation of complement cascades and the opsonisation and lysis of spirochetes [33]. Such complement-binding interactions, together with the down-regulation of immunogenic proteins after initial infection, render the survival of the bloodstream despite a strong immune response.

**Fig. 2:**
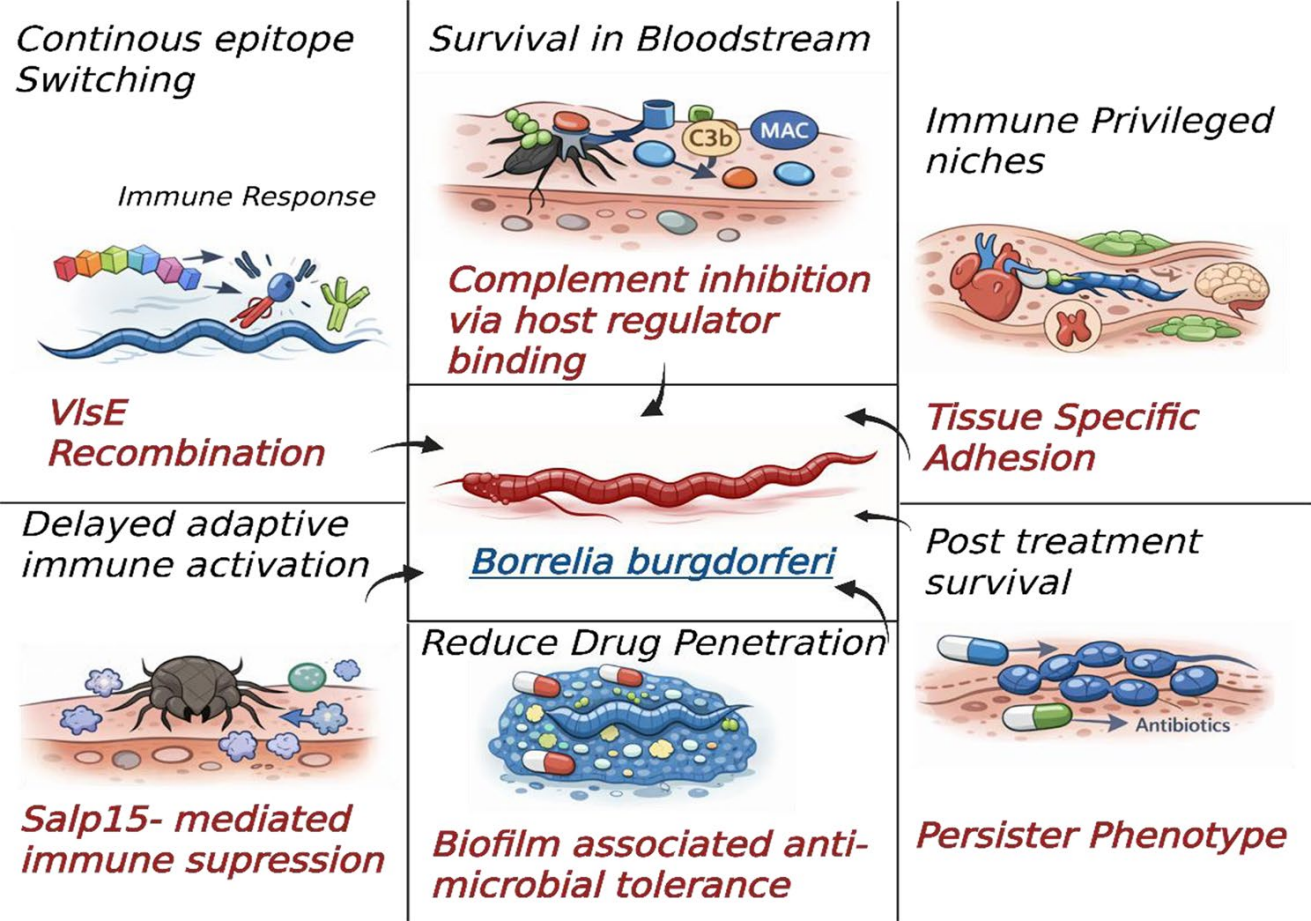
Multilevel immune evasion and persistence strategies employed by *Borrelia burgdorferi* that undermine immune clearance, reduce antibiotic efficacy, and contribute to post-treatment Lyme disease

The immune modulation of the pathogen goes a step further: the salivary proteins of ticks, which are injected into the blood during feeding, suppress signals for local chemokine release and immune cell recruitment into the initial infection site, the dermal tissues, as shown in Fig. 2 [31]. Adaptive mechanisms are also poorly regulated, as infection by *B. burgdorferi* disrupts the germinal centre reaction and antibody development, potentially interfering with a successful humoral immune response against the pathogen.

### Tissue tropism and host-pathogen interplay

*B. burgdorferi* develops tissue tropism due to specific surface adhesins that bind host extracellular matrix (ECM) proteins. Collagen and fibronectin are combined with adhesins (DbpA/DbpB and BBK32) to aid in colonisation of joints and other connective tissues, respectively, that give rise to arthritis and different clinical manifestations associated with Lyme [34]. Premature interaction with ECM also promotes vascular adhesions and transfer to cardiac and neural areas, leading to manifestations such as carditis and neuroborreliosis. Borrelia may be evolving its surface repertoire to suit adaptation to other host niches, as its characteristic adhesin expression patterns vary with the stage of infection. The binding kinetics and molecular determinants of these interactions are still poorly understood, and mechanistic studies can serve as targets for therapeutic disruption.

### Novel: biofilm, persisters and drug tolerance

There is growing laboratory evidence that Borrelia spp. may organise themselves into microcolonies, biofilm-like formations in adverse environmental conditions (e.g. when growing in the stationary phase In vitro) and perhaps in tissues of the host. According to Fabrizio *et al.,* biofilms from *B. afzelii* and *B. garinii* have been linked to an attenuated resistance of the organisms to widely used antibiotics like doxycycline and ceftriaxone in In vitro testing, and these aggregates create extracellular maturity and modified microclimates that may hold back antimicrobial penetration and tolerance [35]. Complementary In vitro and murine models have also reported antibiotic-tolerant, biofilm-associated forms and persister-like forms, which are associated with an increase in the severity of disease or prolonged infection of animal models [36].

Nevertheless, the etiologic relationship between Borrelia biofilms/persister cells and the chronic clinical symptoms in humans remains incompletely elucidated and is under discussion. A large proportion of the supporting data relies on In vitro systems or animal models that are not necessarily representative of human infection, and there are methodological issues (detectivity relative to detection specificity, sampling bias, and variability in experimental biofilm models) that make these difficult to interpret. There are limited and inconclusive clinical data indicating that biofilm-forming or persister phenotypes play a causative role in treatment failure or chronic symptoms in patients. Based on this, although the biofilm and persist-like phenotypical characteristics and sub-cultures are biologically plausible causes of reduced antibiotic efficacy, the hypothesis itself should be offered as tentative: additional studies are required with a standardised in vivo detection system, quantitative antibiotic-penetration and tolerance tests, and properly designed clinical experiments (including longitudinal cohorts and prospective-treatment groups) are needed to determine whether the said phenotypes contribute to human post-therapeutic syndromes [35, 36].

Another, more closely related, dormant subpopulation of *B. burgdorferi* is the persister cells, which can survive high antibiotic concentrations without developing genetic resistance. Conventional studies reported that there were biphasic killing curves in doxycycline-exposed cultures, with a certain proportion of persisters surviving and able to grow again after the antibiotic pressure was removed, indicating the presence of non-replicating drug-tolerant phenotypes [37]. Complementary translational studies have found pathways of regulation (e.g., stringent response regulators), and non-culturable (e.g. viable) states of antimicrobial tolerance during persistent infections [9].

The clinical implication is high. Ordinary antibiotics can sufficiently clear most rapidly growing spirochetes. Still, they might not be enough to deal with persisters and biofilm forms, which may be the reason some patients continue to experience persistent, bothersome symptomatology. This has led to the development of studies on anti-biofilm and anti-persister drugs. Whereas in vitro findings indicate that combinations of membrane-active agents (such as daptomycin) with doxycycline and cefuroxime may be more potent than standard therapies in eliminating biofilm-like *B. burgdorferi*, such findings are, strictly speaking, experimental only and ought to be approached with caution [38]. Daptomycin is an intravenous, membrane-bound antibiotic whose safety profile (increased creatine phosphokinase levels, myopathy, infrequent rhabdomyolysis) is a sensitive issue that must be monitored, particularly when concomitant statin administration is present; it therefore poses feasible and safety constraints to routine clinical application in Lyme disease. Most importantly, the lack of proof or safety of adequately powered clinical trials of a regimen containing daptomycin for human Lyme disease has not been demonstrated, and significant gaps in translation necessarily exist (route of administration, appropriate dosage and PK/PD to support tissue-penetrant activity against biofilm phenotypes, toxicity, and cost). In this regard, such combination regimens must be reported as promising preclinical leads in the manuscript, supporting additional preclinical research and cautious clinical studies, rather than therapies that are ready for implementation in clinical practice [38]. Further studies are examining adjunctive technologies targeting quorum sensing, extracellular matrix degradation, or other metabolic requirements specific to persister states; however, these are largely experimental. Key virulence mechanisms and immune evasion strategies employed by *Borrelia burgdorferi* are outlined in Table 2.

**Table 2:** Immune evasion and persistence mechanisms of *Borrelia burgdorferi*

Mechanism	Molecular components	Biological function	Contribution to persistence/PTLD	Therapeutic implications	References
Antigenic variation	VlsE recombination system	Continuous alteration of surface epitopes	Avoids antibody-mediated clearance, prolongs infection	Limits vaccine and serologic target stability	[30, 31]
Complement evasion	OspE, CspA, CspZ	Binding host complement regulators	Survival in the bloodstream and tissues	Targeting complement interactions may enhance clearance	[33]
Biofilm-like aggregates	Extracellular matrix, alginate-like components	Reduced antibiotic penetration	Increased antimicrobial tolerance	Supports anti-biofilm combination therapy	[10, 35]
Persister cell formation	Dormant, non-replicating subpopulations	Drug tolerance without resistance	Possible contributor to PTLD symptoms	Drives interest in anti-persister drug combinations	[9, 37]
Tick salivary immune modulation	Salp15, Isac, Salp25D	Suppresses local innate and adaptive immune responses at bite site	Facilitates early immune evasion and systemic dissemination	Supports the development of anti-tick vaccines and transmission-blocking antibodies	[31, 39, 40]
Metabolic down-regulation and stringent response	(p)ppGpp-mediated stress pathways	Reduced metabolic activity under hostile conditions	Promotes antibiotic tolerance and survival during nutrient stress	Targeting metabolic regulators may enhance antibiotic efficacy	[9, 37]
Tissue sequestration and immune-privileged niches	Collagen-rich joint tissue, CNS, cardiac tissue	Physical shielding from immune clearance and antibiotics	Contributes to organ-specific late manifestations and persistent inflammation	Enhances rationale for tissue-penetrant or adjunctive therapies	[20, 34, 41]

## Clinical spectrum and coinfections

The course of Lyme disease typically has three significant clinical components: early localised disorder, early disseminated disorder, and late disorder, which demonstrate increasing dissemination of the disease over time, an immune reaction, and involvement of the body. The initial presentation is erythema migrans (EM) - a spreading rash at the site of the tick bite, frequently accompanied by fever and malaise, which occurs days to weeks later and results from infection with *B. burgdorferi* [42, 43]. Without treatment, dissemination via blood and lymphatics leads to the early disseminated stage, which may manifest as multiple secondary EM lesions, lymphocytoma, neurological symptoms (e.g., facial nerve palsy or meningitis), and cardiac manifestations (e.g., atrioventricular block and myocarditis) [42, 44].

Neurologic spread (neuroborreliosis) is characteristic of disseminated Lyme disease and can include cranial neuropathies, radiculoneuritis, and lymphocytic meningitis. Typical examples include Bell’s palsy and sensory impairment, and any CNS malfunction may be due to encephalitis or cerebellar ataxia (in more extreme or unusual instances) [41, 44]. Although Lyme carditis is not always prevalent (it occurs in a tiny percentage of untreated patients- 1% to 10% of untreated cases), this condition can be fatal due to conduction abnormalities [45]. It must be detected at the earliest opportunity and treated [42, 46]. Months or years after infection, Late disease may occur as Lyme arthritis, mostly of large joints, most often the knee joint, and as neural symptoms and, in Europe, acrodermatitis chronica atrophicans [42–44].

In addition to the previously well-documented neurologic and musculoskeletal manifestations, atypical manifestations are gaining increasing recognition in the literature and in studies. In Lyme-infected patients, dysautonomia, including symptoms like postural orthostatic tachycardia syndrome (POTS)-like symptoms, small fiber neuropathy (SFN), have been reported to correlate with abnormalities in nerve fiber density and autonomic dysfunction, which are related to persistent pain, sensory symptoms, and cerebral blood flow alterations and uphold a neural influence, peripheral and autonomic, that cannot be utilized by traditional stages [47]. Such unusual sequelae can be part of chronic symptomology and poor quality of life despite the use of typical antimicrobial therapy.

The implications of coinfection with other tick-borne pathogens are clinically relevant. Co-infection with *Babesia microti*, *Anaplasma phagocytophilum*, or other agents transmitted by Ixodes ticks may result in worsening clinical manifestations, a longer disease course, and additional hematologic or systemic symptoms than Lyme disease alone [48]. Coinfected patients tend to have a longer, more severe fever, fatigue, anaemia, or neurological complications; therefore, symptomatic patients in endemic regions should undergo thorough diagnostic evaluation and specific antimicrobial management.

## Current diagnostics and their limitations

### Two-tier serology

The diagnostic standard of choice, as suggested by the CDC and IDSA, remains two-tier serologic testing (an initial enzyme immunoassay/immunofluorescence assay followed by immunoblot confirmation of IgM/IgG) [49, 50]. Although the algorithm is more specific, its less sensitivity in early and localized Lyme disease should be more properly understood as a biological limitation of the algorithm than a technical defect: during the first weeks of infection, antibodies are yet to reach a detectable titre, and sensitivities around 30–40% in the first weeks are consistently observed to be specific [49, 51]. Guidelines cautiously warn that a negative serologic test does not necessarily mean there is no early disease and recommend that clinical diagnosis (i.e., erythema migrans with a compatible exposure history) guide prompt treatment without awaiting serologic conversion [49, 50]. Additional limitations identified are cross-reactivity (such as with Epstein-Barr virus, autoimmune antibodies or other spirochetal infections), leading to a false-positive EIA or IgM immunoblot result [52], and IgG can persist indefinitely, even years after successful treatment, thus limiting the reuse of serology to differentiate between a current and a previous infection. MTTT with two tiers with dual EIAs is better with early sensitivity, although the fact is not able to eliminate problems of staging or treatment-response association [50, 51].

### PCR and culture

Direct detection, including polymerase chain reaction (PCR) and culture, has shown theoretical benefits but is not used effectively in everyday clinical practice. The sensitivity of PCR in blood and cerebrospinal fluid is low because of short-lived and focal *Spirocheteemia*, and greater yields are only obtained in synovial fluid in Lyme arthritis patients [53]. The culture of *Borrelia burgdorferi* is technically complex, slow, and restricted to specialised research labs, and is inappropriate for routine diagnostics [54]. This has led to direct detection techniques being used only as an adjunct to diagnosis, not as the primary diagnostic tool.

### Rare/Novel: harm from unvalidated “direct-to-consumer” tests

There have been increased analytical and clinical issues surrounding the expansion of direct-to-consumer services for non-standard PCR, urine-antigen, lymphocyte-transformation, and multiplex panels for tick-borne infections. The regulatory bodies (FDA, CDC) have cautioned that not all these tests have been shown to be analytically valid and clinically accurate, with a number of these tests giving high test positivity [54, 55]. The implications of these findings are the possible downstream effects, such as a false attribution of non-specific symptoms, inappropriate prescriptions of antimicrobial agents with an adverse-event risk, and health anxiety, which deserve caution rather than liberal use in clinical practice [54]. Although uncontrolled use of poorly validated assays might complicate evidence-based care and antimicrobial stewardship, in this regard, recent expert comments propose closer regulation, standardisation of assay performances, as well as direct clinical-validation demands before commercial implementation of Lyme diagnostic tests [50, 55]. Simultaneously, better communication between clinicians and patients, application of shared decision making, and effective explanation of pre-test probability have been reported to decrease inappropriate testing and increase patient trust, which should be applied to practice in the meantime, as the gaps in regulation and validation issues are resolved [56].

## Diagnostic innovation and multi-omics signatures

### Next-generation serologic assays

Conventional two-tier serologic diagnostic tests for Lyme disease have identified weaknesses, especially in the early phase of the disease, when sensitivity is low due to a lack of antibody development. To overcome this limitation, new-generation serologic systems are increasingly adopting a single-tier ELISA format using multiple recombinant antigens or peptide libraries in a single assay, with the aim of enhancing early detection and maintaining specificity [57, 58]. Other studies report that multiplex or peptide-based ELISAs can detect antibody responses earlier than standard two-tier algorithms, notably in patients presenting with erythema migrans [59]. Unfortunately, even these promising results do not make the majority of next-generation serologic platforms out of investigation. The existing evidence is mostly based on retrospective studies, case-control studies, or small-scale initial clinical studies, and systematic validation in large-scale studies (in various geographic areas and clinical facilities) is very scarce. Table 3 provides a comparative analysis of traditional and emerging diagnostic methods.

**Table 3:** Diagnostic modalities for Lyme disease: summary of strengths and limitations

Diagnostic method	Sample type	Sensitivity (early vs late)	Key strengths	Major limitations	References
Standard two-tier serology (EIA + immunoblot)	Serum	Early: ~30–50%; Late: ~80–100%	Widely available; guideline-recommended	Poor early detection; cannot distinguish active vs past infection	[49–51]
Modified two-tier testing (dual EIA)	Serum	Early: ~6–80%; Late: ~90–100%	Better early sensitivity than standard testing	Still antibody-dependent	[51]
PCR	Blood, CSF, synovial fluid	Blood: ~10–30%; CSF: ~20–50%; Synovial fluid/tissue: ~50–70%	High specificity; detects active infection	Low sensitivity; adjunct use only	[53, 54]
Transcriptomic/proteomic biomarkers	Whole blood, serum	Early: reported ~50–90% in small pilot cohorts; Late: limited data	Antibody-independent; early detection	Limited validation; small cohorts	[58–60]
T-cell-based assays	Whole blood	Early: reported ~40–80% (pilot data)	Detects infection before seroconversion	Limited availability; investigational	[61]
Metagenomic sequencing	Blood, tissue	Variable- high in enriched/tissue samples; low in unenriched blood	Detects pathogen and co-infections	High cost; bioinformatics complexity	[20]
AI-integrated multi-modal diagnostics	Combined clinical and omics data	Reported performance variable (no consensus range); early claims of high sensitivity require external validation	Integrates diverse data streams	Regulatory and validation challenges	[59, 61]

A related application is the construction of multi-epitope fusion proteins, which present a single recombinant expression construct comprising two or more immunodominant and highly conserved Borrelia peptides. This plan aims to decrease the effects of antigenic variability and strain diversity, which improves sensitivity levels at different disease stages [62, 63]. Even though multi-epitope constructs have shown superior performance in preclinical and early clinical trials, effective prospective, multi-centre validation trials must be conducted before these assays can be regarded as fully developed and used routinely.

### Host response biomarkers

Another approach to pathogen detection that circumvents direct pathogen detection is host-based biomarkers. On the one hand, a transcriptomic survey of peripheral blood samples has demonstrated characteristic innate immune and interferon-based transcriptomic signatures of acute Lyme illness, with potential for diagnostic and prognostic applications [58, 60]. Such signatures can be used to distinguish active infection from post-infectious or inflammatory conditions, a major limitation of antibody-based tests.

Biomarkers are even further enhanced with proteomic and metabolomic analyses. Potential applications in pilot cohort studies have shown that metabolomics based on mass spectrometry has detected unequivocal metabolic fingerprints in Lyme disease patients, with preliminary reports suggesting discrimination from controls in exploratory cohorts [57, 59]. Limitations of the translation-to-clinical-use proposition, however, include heterogeneity in cohort size, inter-platform variation, and conventional validation pipelines.

### Lipidomic and glycomic signatures

Lipidomics has received little attention, yet lipid derivatives are biologically relevant because Borrelia relies on host-derived lipids. Distantly disturbed phospholipid and sphingolipid patterns have been reported in serum and cerebrospinal fluid and may have diagnostic utility, particularly in neuroborreliosis. Likewise, the IgG N-glycan method has been used to identify disease-specific glycosylation, referred to as NG. This empirical biomarker is a high-resolution marker for acute infection and recovery monitoring [64, 65].

Despite the promise, validation problems remain significant. The majority of omics research uses small, geographically restricted groups of participants and heterogeneous sampling times. A key requirement in clinical translation is standardised biobanking, longitudinal sampling and cross-platform reproducibility [58, 59].

### AI-Integrated diagnostic pipelines

The machine-learning and artificial intelligence (AI) strategies offer a highly adaptable platform that facilitates the incorporation of heterogeneous diagnostic features, such as clinical attributes, exposure records, metabolomic levels, peptide-binding patterns, and ecological statistics on tick behavior [61]. A number of exploratory studies demonstrate better classification as a result of the use and application of the curated metabolomic or peptide-binding datasets as opposed to the traditional statistical tools [59, 60]. These findings suggest that machine-learning approaches may capture complex multidimensional patterns; however, reported performance metrics are largely derived from internally validated datasets and require independent external validation.

Nonetheless, the existing body of evidence is weak. Most published models are based on relatively small, retrospective cohorts internally cross-validated, instead of externally cross-validated, which makes them more prone to overfitting, especially when high-dimensional omics features are being analysed on small data sets [60]. The assured AI can identify infection before seroconversion must be taken with a degree of caution because there is no prospective study on effective early-stage detection using AI in the diverse population groups and geographic areas. Moreover, the ability to generalise can be limited by demographic, epidemiological, and strain-related variability, and only a limited number of platforms have been prospectively tested on a large scale in clinical environments. Many early-stage AI diagnostics do not live up to regulatory requirements of transparency, explainability, reproducibility and validated real-world performance. Therefore, at present, AI-based diagnostic tools should be regarded as investigational platforms. There is no prospective, large-scale evidence demonstrating reliable detection prior to seroconversion across diverse populations [60].

### Emerging tools

Besides serology and omics, T-cell assays are another new entrance into the diagnostic field. T-cell receptor sequencing and antigen-specific activation assays aim to enable cells to respond to Borrelia antigens, whether or not antibodies recognise them. T-Detect Lyme is an investigational platform currently in clinical trials and could be used as a supplement to serologic tests, especially in early or atypical disease [61].

Metagenomic sequencing methods enable unbiased identification of Borrelia and co-infecting tick-borne pathogens. In low-biomass samples, capture-enrichment sequencing (e.g., TBD Cap Seq) and shotgun metagenomics have enhanced sensitivity and uncovered clinically relevant coinfections that can elevate disease severity and response to treatment [20]. Though costs and bioinformatic complexity remain obstacles, advances in sequencing technology and targeted enrichment methods are gradually making clinical diagnostics a possibility.

It has been noted that even though there is a buzz when multi-omics platforms incorporating artificial intelligence are implemented as the next-generation diagnostic platforms, assertions relating to high sensitivity or earlier signs, are to be taken with considerations. The majority of the research published is mostly exploratory in nature with a rather small sample size, geographically limited cohort with heterogeneous definition of the cases. Lack of consistency in the geographic distribution of Borrelia genospecies, immunity of hosts, time of samples collection, and pipelines to analyze them reduces cross-study consistency and style. Moreover, most machine-learning models use internal validation methods as opposed to external validation in independent studies thereby enhancing the chances of over-fitting and exaggerated performance samples. Before biomarker panels can be used in clinical practice, standardization is required, computational processes must be harmonized, multicenter validation on a prospective basis, and regulatory evaluation undertaken. Multi-omics and AI-based diagnostics can currently be viewed as an experimental technology and not a proven alternative to the existing and proven serologic testing.

## Treatment: standard of care and controversies

The clinical use of antibiotics is the backbone of the management of Lyme disease, with the treatment plan depending on the stage of the disease and the organ involved. Oral doxycycline is the first-line agent because of its high efficacy, good tissue penetration, and activity against common coinfections such as *Anaplasma phagocytophilum* in cases of early localised and uncomplicated disseminated disease [66, 67]. Cefuroxime axetil can also be used in patients intolerant to doxycycline (or have contraindications), and its efficacy can be compared with that of doxycycline in the treatment of early disease [66]. Ceftriaxone should be administered intravenously; it is preferred over oral ceftriaxone in severe manifestations, especially Lyme neuroborreliosis, high-grade atrioventricular block, or difficult-to-treat Lyme arthritis [66, 68].

The evidence base that underlies these regimens is based on some randomised controlled trials (RCTs) and extensive observational studies. Early studies had determined that doxycycline (10-21 days) or beta-lactams are effective in short courses (10–21 days) in the early stages of Lyme disease, and there is little evidence of protracted treatment [67]. In neuro-manifestations, IV ceftriaxone has been shown to enhance neurological and cardiac outcomes, with no disappearance of symptoms with microbiological treatment in some patients [68].

PTLD Post-treatment Lyme disease (PTLD) refers to a clinical manifestation of incomplete or recurrent symptoms - the most frequently severe fatigue, cognitive impairments, and musculoskeletal pain- that persist after completion of the recommended antibiotic therapy for reported Lyme disease. Several good randomised controlled studies (especially Klempner et al. and Fallon et al.) have tested the use of the extended or repeated courses of antibiotics in the treatment of this condition, and all have shown no long-lasting clinical import or advantage over placebo and have reported higher risks of serious adverse events (line-associated infections, Clostridioides difficile colitis, drug toxicity) [69, 70]. Further European studies came to the same conclusion; they found no lasting positive effect on health-related quality of life, even with long-lasting antibiotics [71]. These excellent RCTs are the main evidence base that these international guidelines are presently basing their empirical advice against routine long-term antibiotic treatment when there is no objective proof of ongoing infection [66].

Notably, there is growing evidence that persistent symptoms cannot be attributed solely to ongoing active Borrelia infection in the vast majority of patients treated. The suggested non-infectious causes include immunologic dysregulation, tissue residual damage, neuroinflammation in the central and peripheral regions, automatism, and changes in pain processing [68, 71]. Animal models indicate that the remnants of non-viable bacteria would trigger prolonged inflammatory reactions, whereas the human studies indicate very few indications of culturable organisms in the presence of the relevant therapy [68, 71]. Such observations resonate with the shift in the end of antimicrobial escalation as an alternative to symptom-focused and rehabilitative approaches to PTLD, such as neurocognitive rehabilitation, pain management, and autonomic dysfunction management.

## Post-treatment Lyme disease (PTLD)/persistent symptoms

### Burden, prevalence, and symptom clusters

**Fig. 3:**
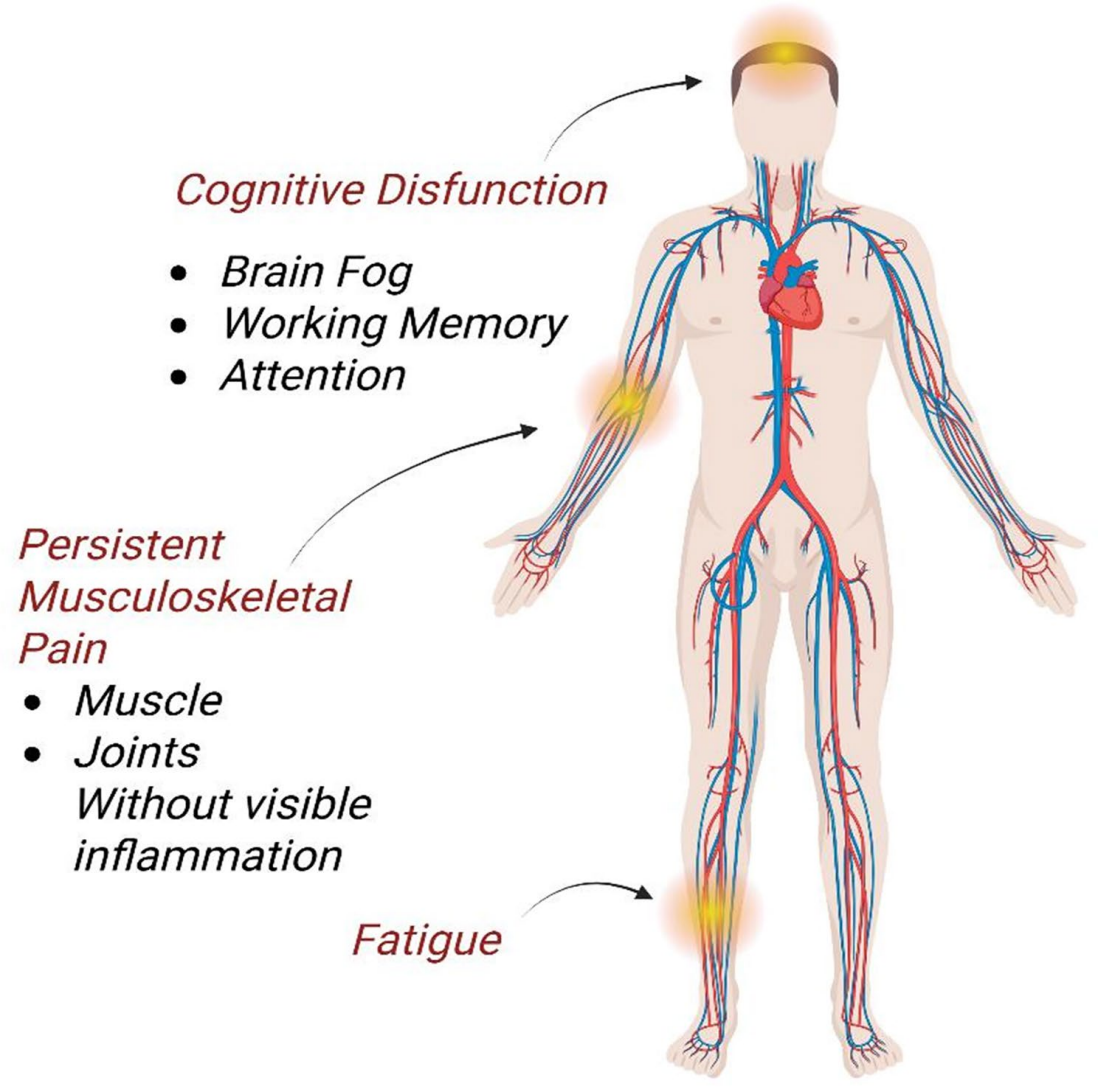
Key clinical features of post-treatment Lyme disease (PTLD)

The post-treatment Lyme disease (PTLD) burden is clustered around three symptom groups, including fatigue, cognitive dysfunction (attention, working memory, and brain fog), and inexplicable ongoing musculoskeletal pain, which do not manifest inflammation, and these are the main sources of functional impairment in cohort studies. In the most extensive prospective study, 27.2% of treated Lyme patients had persistent symptoms at 12 months, fatigue, cognitive impairment, and pain being more pronounced than in the population background prevalence ≈21–23%). Symptoms of the new onset were prevalent at 6 months in a focused prospective cohort: 36% were fatigued, 45% had neurocognitive problems, and 20% widespread pain, which demonstrated significant morbidity in the short term among a subgroup of patients. Clustering of symptoms studies indicate that sleep disturbance and pain are strongly related to fatigue and cognitive complaints, and cognitive impairments measured on formal testing (processing speed, attention, working memory) are correlated with self-reported brain fog. Mechanistic and clinical reviews highlight that such clusters of symptoms are common with no objective markers of inflammation (e.g. synovitis) and argue in favour of models of post-infectious dysregulation (immune, neuroinflammatory and central pain-processing changes). The combined analysis of these data suggests that the cumulative burden following treatment is symptom-concentrated in fatigue, cognitive dysfunction, and non-inflammatory musculoskeletal pain, and the data should be targeted with phenotype and cluster-specific interventions, as shown in Fig 3 [72–76].

Post-Treatment Lyme Disease (PTLD): Clinically significant and lasting fatigue, cognitive impairment, or musculoskeletal pain that continues ≥ 6 months following antibiotic treatment per guidelines for established Lyme borreliosis and reasonable fundamental dismissal of various diagnoses. In the literature, the prevalence estimates tend to be in the 10–20% range, but again eventually depend on the case definition, the date of follow-up, and study design; prospective cohorts with a research-grade case definition typically report less conservative prevalence estimates than convenience samples or surveys of patients themselves [74, 76]. In multiple cohorts, objective deficits that were consistent with functional impairment are supported by formal neuropsychological testing and are the dominant symptom clusters of severe fatigue, objective impairment, subjective impairment (attention, working memory, processing speed), and diffuse musculoskeletal pain [77].

Randomised controlled trials (RCTs) of high quality take up the highest level of therapeutic evidence. Two controlled trials by Klempner MS showed no sustained effect of long (90-day) courses of antibiotics compared with placebo in patients who had received sufficient treatment earlier [69, 78]. Follow-up RCTs by Fallon BA and Berende A also did not demonstrate sustained clinical benefits of extended antibiotic courses and reported higher rates of severe adverse events [70, 71]. Those RCTs thus constitute the most significant evidence base underpinning modern guideline recommendations (the IDSA guidelines), which discourage routine long-term antibiotic retreatment without objective evidence of persistent infection [71].

However, methodological issues with these RCTs are valid and should be mentioned. Critics observe that the inclusion and exclusion criteria were strict, which may have excluded clinically heterogeneous patients who are experienced in practice (e.g., patients previously with multiple coinfections, with atypical serologic phenotypes, with variable objective results), which could limit external validity. Some of the trials were underpowered in important subgroup analyses; primary endpoints were global quality-of-life measurements rather than functional outcomes in specific domains; and treatment regimens were administered homogeneously, which does not necessarily reflect the varied clinical practice (combination antibiotics or biomarker-based, biomarker-guided therapy). This creates the risk of Type II error in small, well-defined subgroups and emphasises Type I error across all biologically defined subpopulations, nullifying the benefit, not by trial results of a narrow false null hypothesis.

Alongside RCTs, there is prospective observational cohort and clinic series evidence of lower level, which records the symptomatic improvement in the chosen patients following the long or combination antibiotic approaches; such information is hypothesis-generating, but is vulnerable to the bias of selection, confounding and unblindedness [79]. Mechanistic tests of animal models demonstrate the fighting ability of non-viable flowers of Borrelia and independent local immune reaction following exposure to antibiotics, which offers a biological possibility of persistent inflammatory sequelae even in the presence of inseparable viable organisms [77]. New human biomarker and transcriptomic literature can provide earlier indicators that can possibly allow stratification and subsequent selective trials [80].

Collectively, the evidence suggests high-quality RCTs do not favour routine extended antibiotic use on unselected patients with PTLD. Observational evidence and non-mechanism data suggest open recognition of current debate and specific studies on some of the prespecified subgroups, biomarker-based, adequately powered randomised trials and non-antibiotic, mechanism-based symptom-guided treatments (neurocognitive rehabilitation, pain control, autonomic therapies) are a higher priority. This framing is balanced and evidence-level, yet responds to the existing guideline positions directly and specifically responds to the methodological criticisms presented by reviewers and clinicians [81].

### Neuroinflammation and central nervous system biomarkers

Neuroimaging has shown that it has similar characteristics of putative neuroinflammation in PTLD. Translocator protein (TSPO) PET imaging of positron emission tomography has shown a positive signal of glial and microglial cells in brain of patients to have been increased following the use of antibiotics, giving in vivo evidence of the presence of neuroimmune activation in a number of cohorts tested [11]. Diffusion tensor imaging (DTI) has shown that white-matter microstructure, especially frontal areas of the executive functioning and working memory, has changed, and multimodal studies have observed structural changes correlate to the objective cognitive impairment in some series [82]. Alterations in the patterns of cytokine and chemokine in patients with neurologic manifestations subsequent to Lyme disease have been characterised by complementary cerebrospinal fluid (CSF) analyses [83].

Nevertheless, these modalities are research modalities and cannot be used as a standard mode of clinical diagnosis. The main shortcomings are the small and frequently single-centre cohorts, heterogeneity of imaging protocols and TSPO ligands, lack of specificity of TSPO binding to specific neuroimmune processes, inconsistency in methodology and analysis pathways in DTI studies and heterogeneity and overlap of CSF biomarker patterns with other inflammatory CNS diseases. There are no standardised acquisition protocols, prospectively validated biomarker thresholds, multicentre replication or a display of clinical utility at present. Due to such reasons, PET, high-end MRI, and CSF profiling should be used only as hypothesis testing, mechanistic investigation, and research-based patient stratification [83].

### Neurorehabilitation and digital therapeutics

Since long-term antibiotic interventions have shown little provable value with risks, non-antibiotic care methods for neurological impairment have gained attention. Cognitive rehabilitation interventions -structured memory-retraining and executive-function therapies-have also been found to have potential in small trials in treating a fraction of patients with PTLD who have objective deficits to the extent of everyday cognitive functioning [84]. Nevertheless, available data are to date based on pilot and uncontrolled studies; randomised, sufficiently powered, and standardised outcomes to determine efficacy, benefit persistence and optimal patient selection are penultimate. The purpose of these interventions will be to develop neuroplasticity and functional compensation and not infection.

Likewise, transcranial direct current stimulation (tDCS) and transcranial magnetic stimulation (TMS) are also under consideration as experimental therapies. There are early clinical trials and pilot studies underway to determine whether noninvasive cortical stimulation can improve fatigue or cognitive manifestations by re-regulating the activity of neural networks, although there is a lack of evidence to support clinical effectiveness, dose-response associations, and long-term safety. Such sensing and digital-health systems can provide objective, continuous monitoring of autonomic functionality, sleep, and activity, and when used with algorithmic symptom monitoring, can be used to optimise outcome measures and make individualised care possible [85]. However, such digital therapeutics and monitoring solutions are currently under investigation: clinical validation, regulatory approval, and the effect on patient-centred outcomes of these solutions need to be established in a prospective study based on rigorously designed clinical trials before they can be suggested as the standard care.

## Emerging therapies and treatment pipeline

### Vaccine development for Lyme disease

In the current state of clinical development, Pfizer and Valneva have developed the multivalent VLA15 vaccine candidate as the most advanced Lyme disease vaccine. VLA15 focuses on *Borrelia burgdorferi*, which causes Lyme disease, and has 6 OspA serotypes that it tries to induce protective antibodies that prevent the spread of the bacteria by infected ticks into humans [3, 86]. In 2023, Pfizer and Valneva reported that they had initiated the randomised, placebo-controlled Phase 3 VALOR trial expected to recruit around 6000 participants aged five years and above in sites in highly endemic regions such as the U.S., Sweden, Germany, Finland and the Netherlands [87]. Participants are given three doses of VLA15 or placebo, followed by a booster, and the trial is followed through to the 2025 tick season to assess clinical outcomes. On July 17, 2024, Pfizer and Valve announced the completion of the primary three-dose vaccination series in the VALOR trial, the main step before efficacy analysis begins [87]. It is anticipated that Biologics License Application (BLA) submissions to the FDA and Marketing Authorisation Applications to the EMA will be submitted in 2026, assuming success in achieving these results. Though VLA15 is a protein-based strategy VLA15 is a protein-based approach, efforts have been made into an mRNA-based multivalent vaccine to encode multiple Borrelia antigens as an adjunct to provide the diversity of strains and flexible production, but these remain in the preclinical phase [88].

### Anti-Tick Vaccines: a novel preventive strategy

Targeting tick salivary proteins essential for feeding and Borrelia transmission, rather than the pathogen or the vector, vector-targeted vaccines represent a new paradigm for preventing Lyme disease. Salp15 is one of the most studied tick salivary proteins. This 15-kDa Ixodes salivary protein binds the outer surface protein C (OspC) of *Borrelia burgdorferi* and protects against complement-mediated lysis and host immunity during the initial infection [39, 40]. A seminal study demonstrated that antiserum raised against Salp15 could protect mice against *B. burgdorferi* upon tick infestation, reduce spirochete loads in skin and heart tissues, and enhance the efficacy of pathogen-directed vaccines targeting classical Borrelia antigens, including OspA and OspC [39]. A separate paper using adenoviral vectors expressing several salivary gland proteins (Salp15, Salp25A/D and Isac) demonstrated that immunisation had the potential to partially control *B. burgdorferi* infection in murine models, which supports multi-antigen anti-tick vaccine constructs [89]. Other novel salivary proteins with altered expression in the feeding and infection states were identified in a quantitative proteomics analysis of *Ixodes ricinus* saliva, which further revealed candidate antigens that, after developed as vaccines, reduced tick engorgement weight and could potentially interfere with feeding success [90]. Taken together, these peer-reviewed results demonstrate that tick salivary proteins are viable vaccine targets for future Lyme disease prevention strategies.

### Strategies targeting persister cells and biofilms

Biology of *Borrelia burgdorferi* persisters and biofilm-like aggregates need a close, contextual presentation and not a direct causal explanation for the continuing human symptoms. *B. burgdorferi* are pleomorphic under stress, and contain round bodies, membrane blebs and matrix-associated aggregates; proposed survival strategies, the interrelationships between these forms and their functions are yet to be fully clarified. In vitro experiments indicate that growing cultures that are at the stationary phase can be used to enrich antibiotic-tolerant subpopulations (persisters) which survive otherwise bactericidal exposures and can regrow on removal of the drug pressure, which supports a tolerant phenotype rather than the acquired resistance phenotype [37]; experimental studies also show that growth may also occur after antibiotic withdrawal in line with the models of In vitro persistence [9]. Ex vivo and tissue publications report matrix-associated aggregates containing putative biofilm markers (such as an alginate-like material), which likely impede antimicrobial penetration and immune access [10]. Drug-repurposing screens have identified agents (daptomycin, clofazimine, cefoperazone) and combinations of three drugs with increased activity in vitro against tolerant phenotypes, and some combinations of three drugs have eliminated persister cultures in laboratory assays [91, 92]. Nevertheless, hasty generalisation to clinical applicability: detection techniques and biofilm biomarkers are inconsistent, animal model data are sparse and non-homogeneous, and in vitro potency is not translatable in the absence of rigorous PK/PD, tissue-penetration, toxicology, and safety studies. IV medications like daptomycin have monitoring needs and have trouble with being toxic, limiting their immediate clinical utilisation. And on this basis, the persister and biofilm concepts are to be understood as mechanistic hypotheses, with no known laboratory/little in vivo studies, but rather as priority research directions as standardised in vivo detection assays, validated animal models, efficacy of drug, and critically designed early-phase clinical studies that combine pharmacology and safety endpoints [91, 92].

### Monoclonal antibodies and passive immunisation

A new potential treatment and prevention method for Lyme disease is monoclonal antibody (mAb) strategies, which offer immediate, specific immunity and do not require the development of a host response. Preliminary experiments revealed that *Borrelia burgdorferi* monoclonal antibodies that specifically bind the outer surface protein A (OspA) of *Borrelia burgdorferi* could prevent infection in immunodeficient mice, and thus passive immunisation could prevent early attachment and disease pathogenesis of the spirochetes [93]. Likewise, mice were protected against OspC antigen-carrying ticks using monoclonal antibodies, demonstrating that immunity targeting the essential surface proteins can block natural vector transmission. [94]. Even more recent research produced human monoclonal antibodies (HuMabs) targeting OspA with potent In vitro borreliacidal efficacy across multiple Borrelia genospecies and protected against tick-borne challenge in murine models, suggesting the effectiveness of preexposure prophylaxis [95]. The development of mAb engineering has resulted in extended half-life forms (e.g., 2217LS) that are effective in protecting nonhuman primates against tick-transmitted infections with a single dose, demonstrating duration and breadth of protection across species such as *B. garinii* and *B. afzelii* [96]. They can be applied on a seasonal basis or after exposure to the disease to prevent it in high-risk populations through passive immunisation. They can be viewed as a worthy addition to active vaccination. Nevertheless, safety, dosing, and long-term efficacy are essential aspects that cannot be achieved without clinical assessment before their general use. Current treatment options and emerging therapeutic strategies targeting Lyme disease persistence are summarized in Table 4.

**Table 4:** Emerging preventive and therapeutic strategies for Lyme disease

Strategy category	Target	Mechanism of action	Disease stage	Development stage	Key advantages	Major challenges	References
Multivalent OspA vaccine (VLA15)	*Borrelia* spirochete	Induces anti-OspA antibodies blocking tick-to-human transmission	Prevention	Phase 3 clinical trials	High specificity; first late-stage human vaccine	Long-term efficacy and coverage across strains	[3, 86, 87]
Anti-tick vaccines	Tick salivary proteins (e.g., Salp15)	Interferes with tick feeding and immune modulation	Prevention	Preclinical	Broad protection against multiple tick-borne pathogens	Complex tick biology; ecological variability	[39, 89]
Anti-persister drug combinations	Persister cells/biofilms	Multi-drug eradication of tolerant bacterial forms	Persistent/PTLD	Preclinical/In vitro	Addresses antibiotic tolerance	Safety, clinical translation	[37, 38, 92]
Reservoir-targeted oral vaccines	Rodent reservoirs	Interrupts enzootic transmission cycle	Prevention (population level)	Field trials	Reduces human exposure risk	Implementation logistics	[97, 98]
Monoclonal antibodies	*Borrelia* surface antigens	Passive immunisation and spirochete neutralisation	Early infection/prophylaxis	Preclinical	Rapid protection in high-risk exposure	Cost; limited duration	[40, 88]
Host-directed immunomodulators	Host inflammatory pathways	Reduces immune-mediated tissue damage	PTLD/late disease	Experimental	Symptom-focused management	Risk of immunosuppression	[41, 68]
Tick microbiome engineering	Tick gut microbiota	Alters vector competence for *Borrelia*	Prevention	Experimental	Innovative One Health approach	Ecological and regulatory concerns	[5, 25, 28]
Anti-microbiota (host) vaccines	Tick-associated microbiota/keystone midgut taxa	Host immunisation elicits antibodies that perturb the tick midgut microbial community, reducing Borrelia colonisation and vector competence	Prevention (population/One-Health)	Preclinical/experimental (animal models)	Mechanistically distinct from paratransgenesis; potentially scalable via host vaccination; translationally attractive within One Health framework	Demonstration of effect in wild tick populations, ecological off-target effects, delivery strategy (target host species/humans), regulatory and ethical considerations	[26, 27]

## Vector control and ecological approaches

### Integrated vector management

One Health viewpoints acknowledge human, animal, and environmental health to be interconnected, a tenet that forms the basis of the Lyme disease through Integrated Vector Management (IVM). IVM integrates mutually supportive plans such as acaricide treatments, host control (e.g. deer exclusion or reduction), landscape and habitat manipulation, and specific use of biological control agents to reduce deer population and stop the *Borrelia burgdorferi* cycle [96, 99]. Acaricides delivered to plants or hosts can result in quick decreases in host-seeking tick densities in managed field trials; nonetheless, within huge randomised trials, the effect on human tick-borne disease occurrence is contingency-based and complemented on practiced faltering times, spack publicity spent, and recolonisation relationships, and carry-over consequences are highly significant [95]. In the same way, the local tick population has been reduced through vegetation management and targeted host-treatment (such as rodent-targeted topical acaricides or deer-directed 4-Poster devices), although the expense, scaleability, or permanence of each method, and each landscape structure, differs [100, 101].

Many studies have found a lowering effect of deer control on using prevailing Borrelia infection in ticks and on the incidence of human disease in a system when interacting with other control tools instead of alone; thus, it is maximum in combination with other interventions [102]. Community involvement, organised monitoring of tick infestation, and infection rates, and adaptable response to local ecology and resource capability are all the characteristics of true IVM programs, which, nevertheless, are appropriate actions that comply with the principles of One Health in ensuring the stability of ecosystems and the reduction of human disease risk [103]. Good IVM is therefore context-planning, constant entomological and epidemiological monitoring e.g. routine and expected outcomes and an iterative process with local data and not a pre-programmed standard [96].

### Reservoir-targeted oral vaccines

One ecological strategy to prevent the spread of Lyme disease focuses on the wild reservoirs of *Borrelia burgdorferi*, particularly white-footed mice (*Peromyscus leucopus*) in North America. Vaccines administered orally through bait stations are intended to elicit protective immune responses in the reservoirs, thereby reducing the likelihood that feeding ticks will contract and subsequently infect in the future [97, 98]. Preliminary field experiments demonstrated that wild mice were vaccinated against recombinant Borrelia antigens, particularly the outer surface protein A (OspA). The prevalence of the enzootic transmission cycle of Borrelia infection in nymphal ticks the following year was significantly reduced [97]. A 5-year field trial assessed oral vaccination of white-footed mice. It revealed that, over 5 years, significant reductions of up to 76% in infected nymph prevalence in a landscape were achieved by oral vaccination of white-footed mice compared to control plots, suggesting that landscape-scale influences on tick infection dynamics and, consequently, on human risk [98]. The concept of modelling using vaccination-effect parameters indicates that oral reservoir-targeted vaccines can effectively reduce Borrelia transmission by vaccinating mice orally against infection, as well as by decreasing transmission from earlier infected/vaccinated individuals [104]. These plans combine ecological knowledge with public health and aim to provide ecosystem-scale control of Lyme disease risk to supplement human vaccination and vector control.

### Environmental interventions

**Fig. 4:**
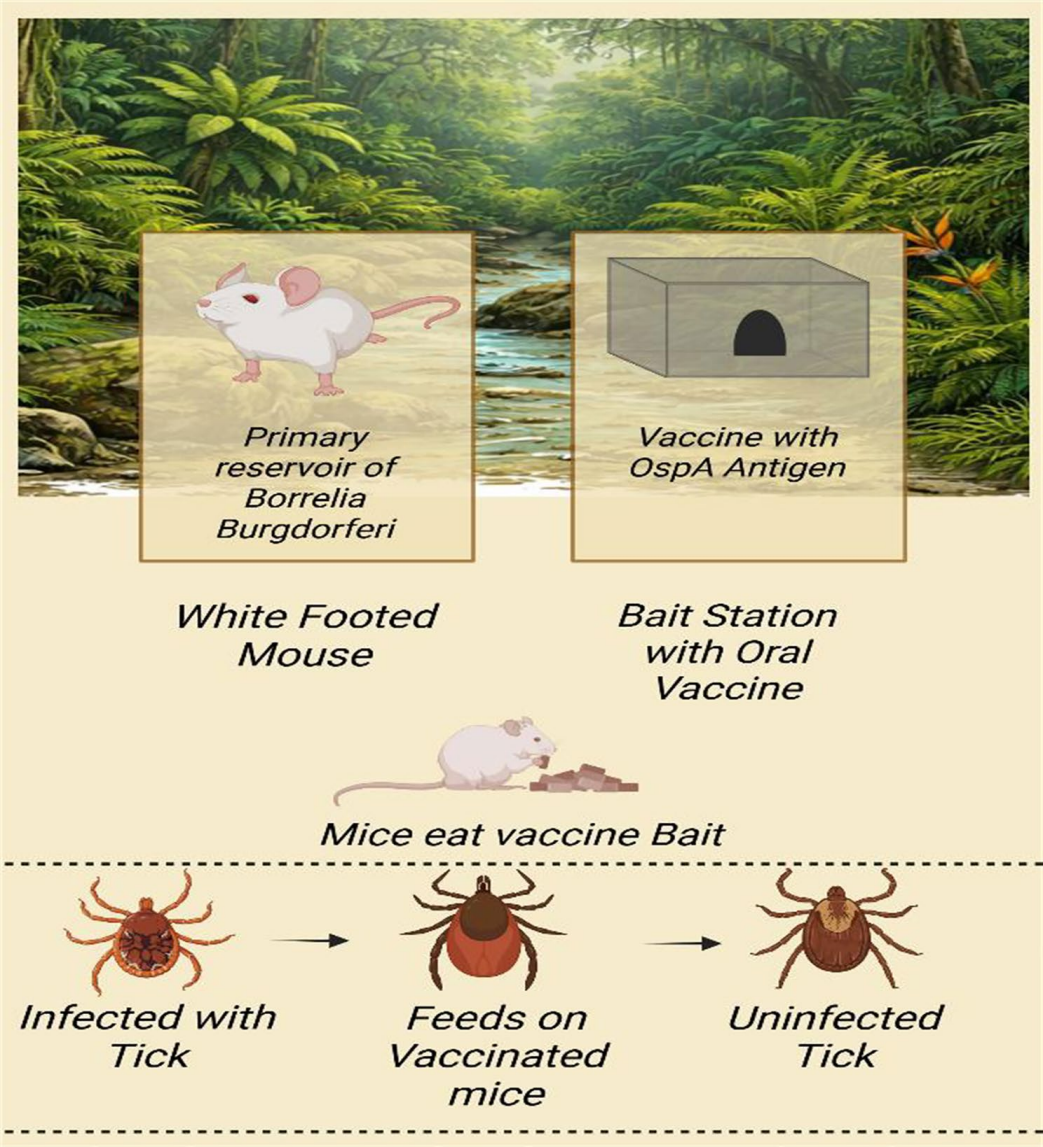
Oral vaccination of white-footed mice reduces *Borrelia burgdorferi* transmission and lowers infected nymphal tick prevalence, decreasing Lyme disease risk

Environmental intervention is an essential element of Lyme disease management in a One Health system that aims to modify the environment to reduce tick populations and to implement surveillance technologies to support specific interventions. Habitat modification includes limiting micro-ecosystems of ticks by clearing litter of leaves, low plants and establishing dry boundaries in the woodlands and leisure zones that decrease the humidity levels that ticks prefer and avoid human fly tick contacts [105]. Combining with surveillance data (e.g., tick density and infection prevalence), such interventions enable managers to focus specifically on the regions to be controlled, making them more efficient and minimising unnecessary environmental impacts, as shown in Fig. 4. New methods use remote sensing and geospatial surveillance to map landscape features, including vegetation cover, moisture gradients, and host habitat distribution, that forecast tick hotspots so that risk forecasting and management planning can be done on larger spatial scales. Environmental health data have also been obtained from remote sensing of land-use changes and climate variables that control tick phenology and host movements, and from matching disease prevention initiatives. These surveillance and ecological practices enable stakeholders to take into account environmental management as a part of community tick control programs, which supports the aim of One Health to minimise the risk of disease due to stewardship of the ecosystems [99, 106, 107].

## Clinical trial design and regulatory considerations

The methodological, clinical and regulatory challenges should be carefully considered designing clinical trials to investigate Lyme disease and post-treatment Lyme disease (PTLD). One of the main problems is in defining meaningful clinical endpoints. Time-to-resolution of symptoms or physician evaluation are also standard Lyme trial endpoints, yet both outcomes are insensitive for assessing persistent or intermittent symptoms of PTLD. Regulatory agencies emphasise the use of patient-reported outcomes and multidimensional functional measures because they are more likely to reflect a treatment benefit, given the real-life consequences of daily functioning and quality of life, rather than merely laboratory outcomes or dichotomous clinical status [108, 109]. Furthermore, the literature indicates that the trials on PTLD have tended to apply heterogeneous inclusion/exclusion criteria, resulting in low generalizability and underpowered outcomes, which means that data-based eligibility models can enhance the representativeness of a trial [110].

Objectivity and longitudinal studies can be enhanced by including biomarkers and digital health measures in trial design. Though no universally accepted biomarker has yet to be confirmed to be true, emerging studies on the topic of metabolic, immunologic and proteomic profiles have shown promise in differentiating symptom patterns and response to treatment, which may be used to guide surrogate endpoints and stratification approaches [111, 112]. At the same time, wearable devices and mobile health applications can continuously record functional status, activity levels, and symptom variability, which can be used in larger datasets to assess the impact of the therapeutic intervention in field settings.

Adaptive designs are flexible for assessing PTLD therapeutics when uncertainty exists about optimal doses or patient subgroups. These designs, example adaptive randomisation, sample size re-estimation, seamless phase II/III designs, enable predefined changes to be made based on interim analyses without violating scientific integrity or regulatory standards [113]. Regulatory directions assist valid adaptive methods after they are thoroughly prespecified, strictly managed, and consistent with proper statistical principles.

## Future prospects of Lyme disease

The future development of Lyme disease research should focus on a translational agenda rather than on expanding descriptive domains. To begin with, discoveries of tick microbiomes need to move toward well-designed, pre-focused ecological studies that assess microbiome stability, non-target effects, and the actual process of affecting the acquisition of Borrelia as a whole, One Health [114]. Second, AI-aided diagnostics needs to go beyond retrospective datasets to large and multicentre prospective validation with predetermined external testing, transparency designed, and performance of clinical usefulness before regulatory incorporation [59]. Third, PTLD studies must focus on highly reproducible, multimodal biomarker development, i.e., a combination of molecular neuroimaging and inflammatory profiling, to facilitate objective patient stratification and validated trial endpoints [11]. Fourth, anti-tick and multivalent vaccines should undergo ecologically informed field tests to assess durability, transmission reduction, and effects on population levels [39]. Fifth, persister-based therapeutic pipelines need to go through a systematic in vivo validation and early clinical pharmacology research to overcome antimicrobial tolerance [91]. Lastly, working One Health modelling, which is underpinned by environmental risk mapping and real-world observation, has to be applied to preventive measures and evaluating the long-term effectiveness of emerging prevention measures in the population [115, 116]. Together, these priorities streamline scientific innovation, regulatory preparedness, and quantifiable public-health value.

## Conclusion

Lyme disease represents a moving population-health perplexity which is considered to be the result of interactions among the ecology of the vectors, biology of *Borrelia burgdorferi*, host immune systems, and ecological alteration. Its relative geographic spreading and dissemination along the Ixodes ticks, reservoir host diversity and land-use patterns indicate the necessity of combined interventions bridging the ecological, molecular pathogenesis, diagnostics, therapeutics, as well as ecology and One Health-based preventive application. In the near-term, sensibly implementable agenda items are enhanced, integrated, localised fusion management programs; enhanced clinician education and diagnostic stewardship; promotion and potential validation of available serologic facilities; provision of standardised clinical trials models in PTLD and adjunctive symptom-based intervention; and standardised clinical trials models. It is based on these steps because they are focused on enhancing and validating the existing tools instead of waiting for the disruptive breakthroughs. Long term objectives in research are transformative innovation: validation of multi-omics-host-response biomarkers and independent maps of acute, persistent and post infectious states; regulatory-grade validation of AI-assisted diagnostic stream; mechanistic elucidation of persister biology and neuroinflammatory pathways so that it can be possible to implement non-antibiotic treatment options; scalable microbiome-based vector control; and operational One Health predictive models that are able to combine risk mapping of the environment with dynamics of transmission. To attain these objectives, multicentre prospective studies, ecological field validation, standardisation of biomarker thresholds and long-term interdisciplinary cooperation will be needed.

The separation between actionable measures and investigational aspirations makes it clear what steps can be taken to translate the translational route to success: short-term benefits will occur through the application of evidence-based enhancements and alignment of the current instruments, but long-term achievements will be seen due to the availability of rigorously validated innovations that would merge the ecological understanding with the strict clinical practice.

## Data Availability

No datasets were generated or analysed during the current study.
